# Development of a predictive model for luteal phase oocyte retrieval in poor responders undergoing natural cycle IVF

**DOI:** 10.1038/s41598-022-11602-0

**Published:** 2022-05-11

**Authors:** Mara Simopoulou, Dionysios Galatis, Evangelos Maziotis, Agni Pantou, Polina Giannelou, Sokratis Grigoriadis, Panagiotis Tzonis, Theodora Griva, Athanasios Zikopoulos, Anastasios Philippou, Michael Koutsilieris, Konstantinos Pantos, Konstantinos Sfakianoudis

**Affiliations:** 1 Department of Physiology, Medical School, National and Kapodistrian University of Athens, 75, Mikras Asias, 11527 Athens, Greece; 2 Centre for Human Reproduction, Genesis Athens Clinic, 14-16, Papanikoli, 15232 Athens, Greece; 3 Royal Cornwall Hospital, Truro, Treliske, UK

**Keywords:** Physiology, Medical research

## Abstract

The aim of this study is the development of a prediction model indicating successful application of Oocyte Retrieval performed during the Luteal Phase (LuPOR) in poor responders, as defined by the retrieval of at least one MII oocyte. Recruitment included 1688 poor responders diagnosed as per Bologna Criteria, undergoing natural cycle ICSI between 2012 and 2020. Oocyte collections were performed during the follicular phase and during the luteal phase similarly. Antral Follicle Count (AFC), Estradiol (E_2_) levels evaluated on both trigger days prior to Follicular Phase Oocyte Retrieval (FoPOR) and LuPOR, and the number of small follicles 8–12 mm that were not aspirated during FoPOR were identified as predictive factors indicative of an efficient LuPOR practice with an Area Under the Curve (AUC) of 0.86, 0.86, 0.89 as well as 0.82 respectively. The combination of the above-mentioned characteristics into a prediction model provided an AUC of 0.88, specificity and a sensitivity of 0.73 and 0.94 respectively and an accuracy of 0.89. The model provided a positive predictive value (PPV) of 93.5% and a negative predictive value (NPV) of 46.8%. The clinical conclusion of the present study aims to be of added value to the clinician, by providing a prediction model defining the POR population benefiting from LuPOR. The high PPV of this model may renders this tool helpful for the practitioner that considers LuPOR.

## Introduction

According to the prevailing theory the female menstrual cycle presents with a single follicular wave. Follicular wave is defined as the synchronous growth of a group of follicles^1^. However, recently this theory has been challenged revealing a second follicular wave in the same cycle^2^. This phenomenon, was first observed in domestic animals such as horses and cattle and thereafter in women^3^. Although these studies have provided newly valuable knowledge, contributing to a better understanding of the physiology entailed, the underlying mechanisms have not been fully elucidated yet^4^.

The second follicular wave has been introduced as an encouraging means towards optimizing the context of in vitro fertilization (IVF) success rates for infertile women and especially for those who present as time-sensitive groups-namely women with poor ovarian response (POR). Poor response to ovarian stimulation for women undergoing IVF presents a standing challenge in assisted reproduction technology (ART) treatment^5,6^.The prevalence of POR has been reported to be between 5 and 35% in infertile women^7^. POR incidence may lead to a significantly low live-birth rate^8^, while 50% of cancelled IVF cycles originate from poor responders’ treatment according to the Society for Assisted Reproductive Technology (SART)^9,10^. As observed, there is considerable range regarding the incidence of POR. This may be attributed to the different definitions of POR. The Bologna criteria were the first concrete endeavor to reach a consensus in the definition of POR^11^, and still remain widely accepted. However, it should be noted that even according to the Bologna criteria, women diagnosed with POR remain a widely heterogenous population. This may be attributed to the fact that POR may be caused by numerous factors, while it is not yet certain if this pathology is present outside the context of IVF^12^. According to a recent study the differential expression of certain micro-RNA pathways is implicated in POR pathophysiology and reflects various subcategories within this population that may enable promising profiling of patients^13^. A successful treatment for POR should consider the woman’s level of advanced age and evaluation of the respective ovarian reserve^6^.

In light of these considerations, and aiming to efficiently address the demanding pathophysiology of POR, new protocols have been suggested, combining conventional follicular phase stimulation (FPS) with luteal phase stimulation (LPS)^14^. In clinical practice the dual ovarian stimulation, known as “DuoStim”, is an interesting approach aiming to maximize the oocyte yield in a shorter timeframe^15^. Numerous studies attempted to compare the developmental potential between oocytes retrieved from follicular phase and those from luteal phase. Their observations have indicated that there are no statistically significant differences in maturation status and fertilization rate of the oocytes, supporting the value of luteal phase oocyte retrieval practice (LuPOR)^16^. Furthermore, in terms of reporting on embryonic development, live birth rates and genetic abnormalities, no statistically significant difference was detected^16–20^. Beyond that, it is worth mentioning that LuPOR practice may further benefit the patient, as the high progesterone and oestradiol levels during this oocyte retrieval act in a protective fashion by preventing the development of a cystic follicle^18^. A cystic follicle is defined as an anovulatory follicle that continuous to develop following ovulation. Moreover, a comparison between the DuoStim and the conventional follicular phase stimulation, known as ConStim practice revealed a higher number of mature MII oocytes, and a greater number of good-quality embryos, favoring the practice of DuoStim^16^. However, it should be noted that LuPOR may present with an added cancellation rate^14^. One should take into consideration that this may be attributed to the identity of the population that LuPOR is commonly applied to, namely women of POR and/or advanced maternal age in which cases a higher cancelation rate may be anticipated de facto.

The rationale of this study stems from the fact that the newly introduced practice of LuPOR is still under investigation despite the promising data sourced to date. In the era of personalized and precision medicine, relying solely on a rather generic diagnosis such as POR to guide practice towards effective treatment may not be enough. As hitherto published data fails to conclusively indicate grounds for decision-making in application of LuPOR, the present study aims to investigate and identify patient characteristics that may indicate successful application of LuPOR, defined as the retrieval of at least one mature (MII) oocyte. The clinical end-point of this study aiming to report back to the practitioner, is development of a predictive model identifying the optimal poor responders’ population benefiting from LuPOR practice.

## Results

A number of parameters were assessed on their prediction capability when considering their relationship with successful LuPOR practice yielding at least one MII oocyte. These factors were: Maternal age, previous unsuccessful IVF cycles, BMI, FSH, LH, FSH/LH ratio, prolactin, progesterone, AMH, AFC, E_2_ levels as recorded on FoPOR and LuPOR trigger days along with the number of small follicles of diameter between 12 and 17 mm. Patient characteristics are presented in Table 1.

**Table 1:** Descriptive statistics of participant general characteristics.

	Mean ± sd	AUC
Age	40.70 ± 1.69	< 0.6
Number of previous stimulated IVF cycles	4.36 ± 1.35	< 0.6
BMI (kg/m^2^)	23.76 ± 4.82	< 0.6
FSH (IU/L)	11.61 ± 6.15	< 0.6
LH (IU/L)	9.43 ± 5.76	< 0.6
AMH (ng/ml)	0.89 ± 0.43	< 0.6
Prolactin (ng/ml)	21.78 ± 5.43	< 0.6
Estradiol on FoPOR trigger day (pg/ml)	260.16 ± 50.01	0.86
Progesterone (ng/ml)	14.35 ± 3.28	< 0.6
AFC	4.78 ± 0.90	0.86
FoPOR number of oocytes retrieved	1.03 ± 0.55	N/A
FoPOR number of MII oocytes retrieved	0.83 ± 0.45	N/A
Number of small follicles	2.71 ± 0.89	0.82
Estradiol on LuPOR trigger day	243.37 ± 49.41	0.89
LuPOR number of oocytes retrieved	1.01 ± 0.40	N/A
LuPOR number of MII oocytes retrieved	0.77 ± 0.42	N/A

**Figure 1:**
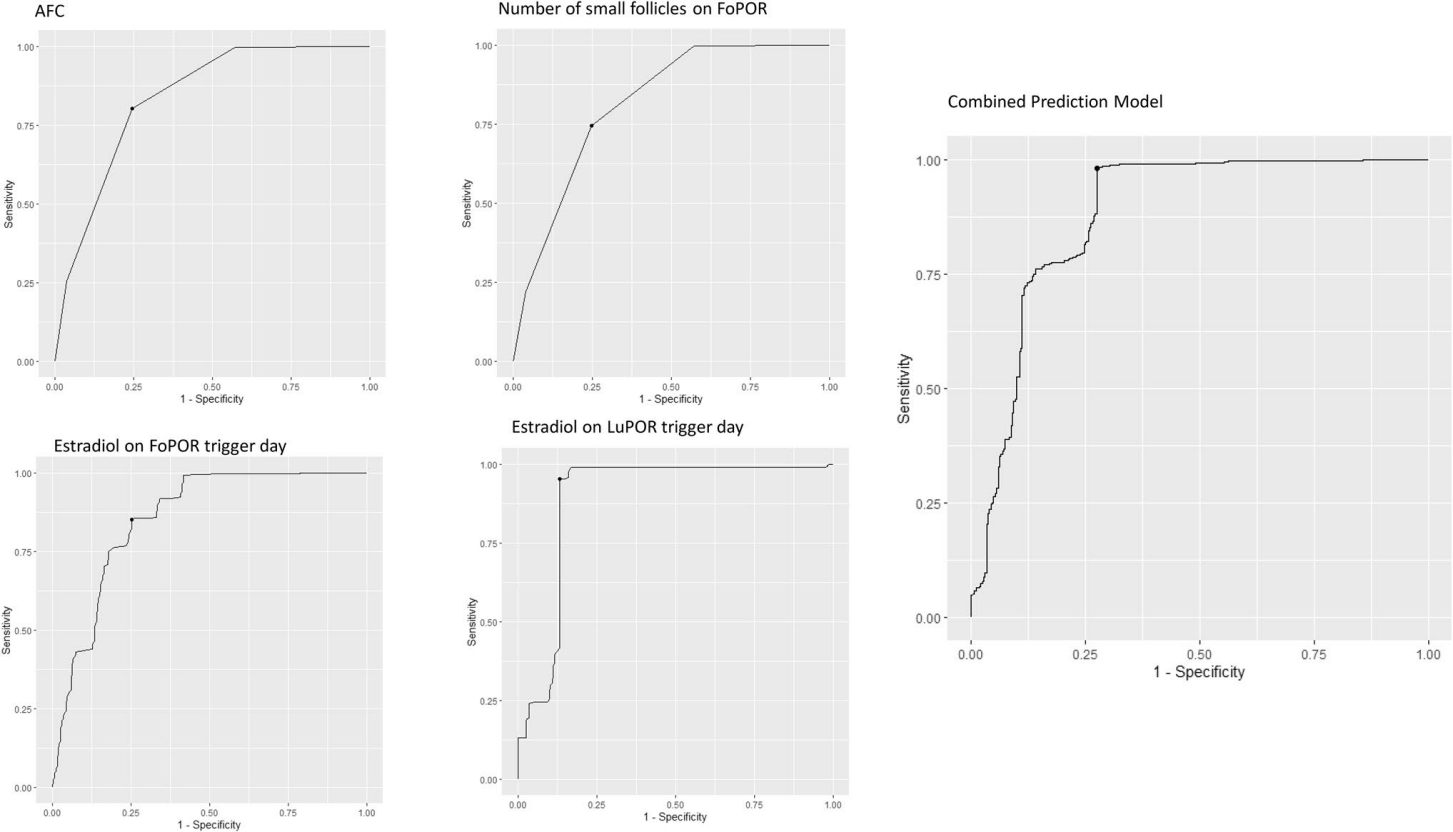
ROC curves of the parameters associated with LuPOR success, and of the combined prediction model.

The factors that were found not to be predictive of an efficient LuPOR practice as the AUC was observed to be below 0.6 were: maternal age, unsuccessful IVF cycles, BMI, FSH, LH, AMH, prolactin and progesterone levels were not predictive of successful LuPOR. AFC as recorded on day 2 of the menstruation period was observed to be predictive of a successful LuPOR with an AUC of 0.86. The sensitivity was 0.8 and specificity 0.75, and the accuracy was 0.79. The optimal threshold value was set at 4.47. E_2_ levels as evaluated on FoPOR trigger day were observed to be predictive of a successful LuPOR and presented with an AUC 0.86. The specificity was 0.75, sensitivity 0.86 and the accuracy was 0.82. The optimal threshold value was set at 232.66 pg/mL. Similarly, E_2_ evaluated on the day of trigger during LuPOR presented with an AUC at 0.89, specificity at 0.85, sensitivity at 0.95 and accuracy at 0.92. The optimal threshold value was set at 200.89 pg/mL. The number of small follicles of during FoPOR also presented to be predictive of the presence of at least one MII oocyte during LuPOR. The AUC was 0.82, specificity was 0.75, sensitivity was 0.76 and accuracy was 0.75. The optimal threshold value was set at 2.94. The combination the above-mentioned characteristics, that were observed to be predictive of successful LuPOR into a single predictive model provided an AUC at 0.88, specificity at 0.73, sensitivity at 0.94 and accuracy at 0.89. The positive predictive value was 93.5, whereas the negative predictive value of the model was 46.8%. The model’s prediction capabilities as well as the ones of the aforementioned parameters are presented in Table 2. The ROC curves of the model and of the aforementioned parameters are presented in Fig. 1. In an attempt to provide a timelier prediction, the authors have proceeded to evaluate whether the combined model could provide accurate predictions earlier. When, excluding the parameter of E_2_ levels on LuPOR trigger day the new model provided a slightly lower AUC at 0.85, specificity at 0.75, sensitivity at 0.87 and accuracy at 0.84. The positive predictive value was 84.4%, while the negative predictive value was 51.5%.

**Table 2:** Prediction capabilities of the included parameters and of the developed model.

	Sensitivity	Specificity	Accuracy
AFC	0.8	0.75	0.79
Estradiol on FoPOR trigger day	0.75	0.86	0.82
Number of small follicles	0.75	0.76	0.75
Estradiol on LuPOR trigger day	0.85	0.95	0.92
Model	0.73	0.94	0.89

A statistically significant difference in cycle cancellation was observed between FoPOR and LuPOR cycles (309 vs. 487 cancelled cycles; RR 1.31; 95% CI 1.23–1.41; *p* < 0.001). When analyzing the cancellation reasons, premature ovulation was responsible for 110 cancellations during FoPOR and 27 cancellations during LuPOR. During FoPOR 126 cycles were cancelled due to failure to retrieve an oocyte, while 73 cycles were cancelled due to retrieving only GV or MI oocytes. During LuPOR 344 cycles were cancelled due to lack of oocytes retrieved, while 116 cycles were cancelled due to retrieving only GV or MI oocytes. Following ICSI procedure no statistically significant difference regarding fertilization rate was observed between oocytes originating from FoPOR compared to oocytes originating from LuPOR (0.74 ± 0.49 vs 0.68 ± 0.48). Similarly, no statistically significant difference was observed regarding the embryo quality on day 3 between embryos originating from FoPOR or LuPOR oocytes. Moreover, no statistically significant difference was observed regarding the blastocyst formation rate between the oocytes originating from FoPOR or LuPOR. The comparison regarding embryo development between FoPOR and LuPOR is presented in Table 3. None of the aforementioned parameters was predictive of fertilization or cleavage rate.

**Table 3:** Comparison of mean ± sd of IVF laboratory outcomes as well as day 3 embryo grading following FoPOR and LuPOR.

	FoPOR (n = 1688)	LuPOR (n = 1688)	*p* value
Oocytes Retrieved	1.03 ± 0.56	1.01 ± 0.40	0.78
MII Oocytes	0.83 ± 0.45	0.77 ± 0.41	0.45
Total number of cycles cancelled	309 (18.31%)	487 (28.85%)	< 0.001
Premature ovulation	110 (6.51%)	27 (1.60%)	< 0.001
No oocytes retrieved	126 (7.4%)	344 (20.38%)	< 0.001
2PN Oocytes	0.64 ± 0.48	0.58 ± 0.49	0.37
Fertilization rate (%)	74% ± 49%	68% ± 48%	0.41
Cleavage stage embryos	0.59 ± 0.49	0.54 ± 0.50	0.38
Top quality	205 (20.69%)	187 (20.37%)	0.95
Good quality	352 (35.51%)	344 (37.47%)	
Moderate quality	289 (29.16%)	251 (27.31%)	
Poor quality	145 (14.63%)	136 (14.80%)	
Total Cryopreserved	0.50 ± 0.47	0.46 ± 0.49	0.63
Number of blastocysts	0.49 ± 0.50	0.45 ± 0.50	0.69

## Discussion

According to the results of our study, AFC, estradiol levels on both trigger days as well as the number of small follicles observed may be predictive of a successful LuPOR. When combining the above-mentioned characteristics associated with LuPOR success in a single model, the resulting model ascertains high accuracy. An AUC of 0.88 is considered to be excellent^21^. When evaluating validation, the results are similar. The model presents with a high positive predictive value of 93.5%. On the other hand, the negative predictive value is 46.8%, meaning that it may not be accurate when employed for excluding women for LuPOR.

Application of LuPOR is steadily gaining ground in the field of ART. Promising data has been published buttressing this practice, albeit questions still remain unanswered, and practitioners may find themselves in a conundrum when considering its employment^22^. LuPOR is one of numerous approaches that may be characterized as a “treatment add-on”. These add-ons should be thoroughly evaluated prior to being included in clinical practice^23^. HFEA’s “traffic light system” is a step in this direction^24^, however the list includes only a limited number of novel approaches. LuPOR and DuoStim have not yet received rating in HFEA’s system, however double stimulation for poor responders has been labelled “research only” in ESHRE 2019 guidelines^25^.

Concurring on the validity of LuPOR practice as an IVF add-on has served as the main driver to conduct this study. Initial employment of LuPOR pertained to emergency fertility preservation^26,27^. Nonetheless, since its conception in early 2010s^28,29^, its application appears to address patients with diminished ovarian reserve and POR. When considering data sourced in this study it appears that LuPOR performs equally well to FoPOR regarding the IVF laboratory outcomes examined herein. When employing the DuoStim protocol, a higher number of retrieved oocytes, higher number of mature MII oocytes, and higher number of available, good-quality embryos are reported when compared to conventional stimulation^14,16^. Moreover, a higher cumulative live-birth rate and a significantly lower time-to-pregnancy are expected when employing the DuoStim approach^14^. Nonetheless, it should be noted that published data presents with limitations and should be interpreted with caution. The most important limitation regarding DuoStim is the lack of any randomized controlled trial to solidify its benefits. It is only conduct of Randomized Controlled Trials (RCTs) that can support use of a novel treatment and enable the shift from experimental to clinical routine practice. Moreover, as reported in literature, DuoStim protocols include the embryo accumulation strategy enabled by the two stimulation cycles performed, hence adding another level of complexity in assessing its clear value. However, all comparisons hitherto include only one stimulation in the control group, hence the embryo accumulation strategy cannot be employed. This diversity between the two management options renders the respective comparison compromised. This type of comparison should be performed prior to concluding on DuoStim’s efficiency^22,30^. Furthermore, a recent meta-analysis in LuPOR, based on cohort studies, reported a significantly high heterogeneity in the results, despite the authors’ attempt to perform a subgroup analysis, thus homogenizing the population^16^. This level of heterogeneity may be attributed to the fact that the POR population is highly heterogenous and possibly DuoStim and LuPOR may not be the optimal strategy nor benefit all POR patients universally. In the present study no statistically significant difference was observed between the number of MII oocytes retrieved in LuPOR compared to FoPOR. This is in agreement with some studies^15^, whereas it seems to be in disagreement with other studies^14,31^. The disagreement with part of the literature may be attributed to the fact that this study employed strictly natural IVF cycles, whereas the other studies employed COS. The rational of performing multiple retrievals during a natural cycle is that a double oocyte retrieval in the same menstrual cycle may enhance the number of oocytes retrieved in an identical time-frame when compared to the conventional single retrieval per menstrual cycle, and this is of added value especially for the time-sensitive group of POR women.

The theory of follicular waves suggests that following each ovulation a second wave of follicles may be recruited, due to a rise in FSH^32^. Following the mid-cycle FSH surge, the first luteal phase wave of follicular development is recruited. However, during the mid-luteal phase, the corpus luteum secretes progesterone that suppresses the pituitary gonadotropins secretion and thereby prevents the dominant follicle selection in this wave^2^. As recently reviewed by Baerwald and colleagues, according to the follicular wave theory, follicles may grow in diameter following the major ovulatory wave even though they may not lead to a second ovulation^32^. In the majority of women, there is only one major wave during the menstrual cycle^2^. In order to override physiology and lead to ovulation, a second triggering was performed for the cases described herein, which allowed for oocyte maturation and retrieval. It may seem that in a natural cycle without hCG triggering, the second ovulation would not be possible. However, it is worth mentioning that women in perimenopause, typically presenting as POR according to Bologna Criteria, may constitute an exception to the aforementioned as a second ovulation has been observed in the same menstrual period, without employing triggering^33^. A different physiological response may be entailed in menstrual cycles encompassing two follicular waves rather than one. As Baerwald recently highlighted, this merits further investigation^32^. In order to be able to delineate this, conduction of further studies reporting on the physiological processes is necessary. Future studies reporting on the transcriptomic profile of these women, rather than the clinical outcomes may shed more light on this phenomenon and provide us with answers regarding the multiple follicular wave theory.

According to our results AFC, evaluated on day 2 of the menstrual cycle is predictive of the retrieval of at least one MII during LuPOR. AFC is a marker of ovarian reserve according to both Bologna and POSEIDON criteria^11,34^. It should be noted that the AFC cut-off value was 4.47, meaning that at least 4–5 follicles should be ultrasonographically identified to indicate a good LuPOR performance prognosis. This number of follicles is similar to the cut-off value for poor-responders according to both Bologna and POSEIDON criteria. Besides the number of retrieved oocytes and their maturity, AFC has been associated with the probability of clinical pregnancy and live-birth^35^ and may serve as a success predicting parameter in a natural cycle regardless of phase. The value and meaning of the number of small follicles identified during FoPOR has been considered in literature to correspond to the value and meaning that AFC respectively holds for LuPOR^36^. It has been reported that follicles of a diameter ≤ 12 mm present with a lower MII oocyte rate when FoPOR is considered^37^. Nonetheless, it may be assumed that not aspirating these follicles during FoPOR, we allow them to continue developing, subsequently resulting to a higher probability of retrieving a mature MII oocyte when employing LuPOR.

Estradiol levels on both trigger days were associated with LuPOR success. E_2_ is involved in the negative-feedback of the Hypothalamus-Pituitary-Ovaries axis, regulating gonadotropin production and selection of the dominant follicle^38^. As it has been observed in animal models, E_2_ influences the number of the follicles, as well as the oocyte size^39^. It has been proposed that a triangle regulation between FSH, AMH and E_2_ is in place during folliculogenesis. FSH stimulates E_2_ synthesis which in turn downregulates follicular synthesis of AMH^40^. During follicular growth E_2_ increases FSH- and LH- receptors expression and thus enables the recruitment process of follicles^40^. In humans an association between E_2_ levels and the number of oocytes and MII oocytes retrieved has been reported^41^. According to results of our study LuPOR E_2_ was the most defining predictor of LuPOR success. This indicates that data sourced from the luteal phase may hold a higher predictive value on events that take place during the luteal phase, in comparison to data sourced from FoPOR, albeit FoPOR data also holds significant predictive value. The levels of estradiol peak mainly 1 to 2 days prior to ovulation. A recent study developed a prediction model for ovulation by summarizing the findings of three other studies, reported average estradiol levels ranging from 180 to 236 pg/ml two days prior to ovulation, which is in agreement with our study reporting a threshold value of 232 pg/ml on the day of trigger^42^. On the other hand, another study employing natural cycles IVF, set the threshold value significantly lower at 101 pg/ml^43^. However, this threshold value was set as a strict cut-off point below which no pregnancies were observed, rather than a predictive threshold value.

An interesting result of the study is the lack of correlation between AMH and the retrieval of at least one MII oocyte, or retrieval of an oocyte regardless of maturity status. AMH is a known marker of ovarian reserve and has been correlated with the number of oocytes and MII oocytes retrieved^11,34^. This lack of association may be attributed to the fact that the study included strictly natural cycles and the main outcome measure could be described as qualitative and not quantitative. Despite the fact that AMH levels are predictive of cycle cancellation in the general population^44^, when restricting the population to strictly poor responders, no association between AMH levels and cycle cancellation was observed^45^. On the other hand, a correlation between AMH levels and number of oocytes and MII oocytes retrieved was reported, in the same population^45^. It may be extrapolated that AMH is a qualitative marker when evaluating strictly poor responders. However, it should be mentioned that in poor responders with very low AMH levels (< 0.5 ng/mL) this extrapolation may be disputed^46^.

The fact that none of the aforementioned parameters was observed to be indicative of the fertilization or cleavage rate is in accordance to literature. It has been observed that AFC, E_2_ , AMH, FSH or LH should not be employed as predictive factors regarding either fertilization or cleavage rate^44,47–50^. Only prolactin levels have been reported in literature to be predictive of fertilization and cleavage rate, with a cut-off value of 16.05 ng/mL^50^. However, in our study the vast majority of patients were above that threshold value, which may be the reason justifying the lack of association^51^. Progesterone levels have been observed to be associated with number of oocytes retrieved and fertilization rate in FoPORs^52^. The lack of association in this study may be attributed to the physiologically high levels of progesterone during LuPOR.

The overall cancellation rate of LuPOR was statistically significantly higher when compared to FoPOR. This is in accordance to current literature^14^. When examining the cancellation due to premature ovulation, it was significantly lower in LuPOR, presumably due to the physiological processes that prevent the second ovulation. Nonetheless when considering cycle cancellation on the grounds of failure to retrieve an oocyte, it appears that in the case of LuPOR a higher number of retrievals resulted to failure to retrieve an oocyte or specifically an MII oocyte. It should be mentioned that the cycle cancellation rate in current literature accounting for all types of cycles is less than 10%^16^. However, in our study the cancellation rate was almost twofold higher (28.85%). This may be attributed to employment of natural cycles which have been generally associated with higher cycle cancellation rates.

**Figure 2:**
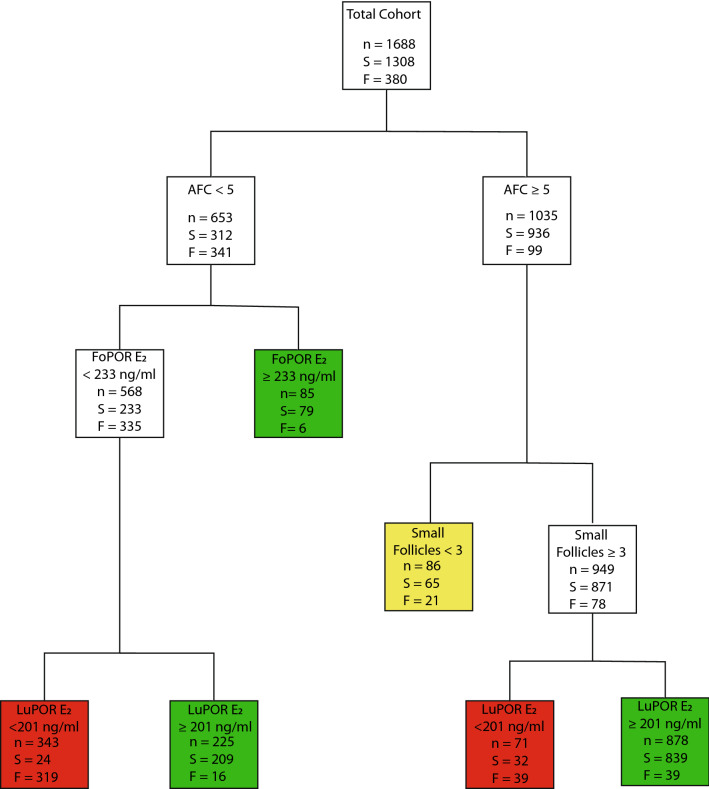
A clinically applicable decision-tree diagram based on the cut-off values and AUC of this model. *AFC*: Antral Follicle Count; *FoPOR E2* : Estradiol levels evaluated on Follicular Phase Oocyte Retrieval trigger day; *Small Follicles*: Number of follicles with diameter less than 13mm recorded on FoPOR; *LuPOR E2* : Estradiol levels evaluated on Luteal Phase Oocyte Retrieval trigger day; *n*: Number of observations; *S*: LuPOR success, defined as the retrieval of at least one MII oocyte;* F*: LuPOR failure, defined as failure to retrieve an MII oocyte. The decision tree is hierarchized according to the timing of each observation. For a node to split, each parent node should consist of at least 100 observations and each child node should include at least 50 observations. If the parent node consisted of more than 100 observation, but one of the child included less than 50 observations, the splitting was performed at the next observed parameter. Terminal nodes are colored. Green color represents good prognosis regarding LuPOR success; Yellow color represents LuPOR success, albeit with lower probabilities compared to the total cohort. Red color represents poor prognosis regarding LuPOR success.

A clinically applicable decision-tree diagram based on the cut-off values and AUC of this model is provided as Fig. 2. The decision tree is hierarchized according to the timings of the observations. The first observation is AFC. It may be observed that with an AFC of 5 or higher, a better prognosis can be anticipated. If AFC is higher than 5, a moderate prognosis may be anticipated when the number of small follicles recorded on FoPOR is less than 3. Further splitting for this observation was not performed due to the small number of women fitting the criteria. When 3 or more small follicles were observed LuPOR E_2_ was the determining factor. It should be mentioned that the vast majority of women at this stage presented with LuPOR E2 levels of 201 ng/mL or more, leading to a good prognosis. Lower E_2_ levels were indicated as a poor prognosis for LuPOR success. When AFC was lower than 5, FoPOR E_2_ levels of 232 ng/mL or more could lead to a good prognosis. For women with lower FoPOR E_2_ levels, LuPOR E_2_ levels should be observed. Women with LuPOR E_2_ levels of 201 ng/mL or more presented with a good prognosis, whereas women with LuPOR E_2_ less than 201 ng/mL presented with a poor prognosis. It should be highlighted that the decision tree includes both the development and the validation set and it stands only as a graphical representation of the results of the model.

While the proposed model presents with significant predictive capabilities, the authors have proceeded with developing an additional model, excluding the E_2_ on LuPOR trigger day. This may provide a timelier prediction for the group of patients that would benefit from LuPOR, relieving the additional waiting time, and psychological burden that patients may experience, as the physician would be able to recommend the course of action immediately following FoPOR. The model excluding E_2_ on LuPOR trigger day presented with lower predictive capabilities in terms of AUC, sensitivity, specificity, accuracy and positive predictive value. However, the prediction status remained at a very good level (AUC: 0.85), while the negative predictive value was higher (51.5% vs 46.8%). Despite its slightly reduced predictive capabilities, it may be of added clinical significance providing an initial screening of the patients and assisting clinicians in decision making and planning of treatment course.

Employment of natural cycles may present both as a strength and as major limitation when examining the value of this study. It may be possible that the cut-off values reported herein may be altered when COS is employed. However, the parameters that do present an association with LuPOR success should be taken into account when considering any scenario of LuPOR practice. Moreover, when employing COS, dosage and duration of stimulation should be evaluated. Since internal validation may be confounded by the fact that this was a single center study, an external validation to solidify the results of the present study is required. In addition to this, a future model would benefit from including levels of Inhibin-B in the analysis as it has been reported to be of high biological significance in the folliculogenesis process^53^. Another limitation of the present study is the relatively low negative predictive value. This could be compensated by maximizing specificity or negative predictive value when setting the cut-off values. It may be possible that maximization either of the two aforementioned metrics would result in a higher negative predictive value. This could result in lower number of retrievals failing to yield oocytes. However, this could affect the sensitivity and positive predictive value, which could subsequently lead to a fewer accumulated oocytes for this time-sensitive group of patients. Thus, the authors have employed maximization of Youden’s index attempting to present an equally high sensitivity and specificity. It should be highlighted that the predictive value of E_2_ regarding successful LuPOR application may be biased by the fact that it was employed as an indication for performance of LuPOR. However, the cut-off value employed as an indication is significantly lower compared to the cut-off value that was identified as predictive of a successful LuPOR application. Another possible limitation of the study is the fact that during FoPOR follicles sized between 8 and 12 mm were not aspirated according to the Clinic’s Standard Operating Protocols. The reason behind this is that aspirating smaller than 12 mm diameter follicles may result in yielding an immature oocyte. Specifically, it has been reported that only 1 in 4 follicles of that diameter results in an MII oocyte^54^. On the other hand, when such follicles are left to be aspirated during the luteal phase this may result in yielding mature oocytes, and this served as the rationale behind this practice. Regarding the strategy of double oocyte retrieval, further studies are required to determine the minimum required follicle diameter for aspiration during FoPOR, as various cut-off values have been employed in literature^31,36^.

Further to these limitations it should be noted that despite the fact that solely women with POR diagnosis were included, population variation cannot be excluded as a possible reason for caution. It may be possible for a number of women POR diagnosis may be a result of gene variations. It may be of importance in a future study to evaluate the practice of LuPOR in women with specific genetic causes of POR. Further to this, especially regarding the successful fertilization and cleavage outcomes, it may be possible that a number of factors that have not been studied may have influenced the results of the present study. Confounders posed by ICSI application are adding another level of complexity in assessing the oocyte’s performance. What is more male factor infertility has been associated with poorer fertilization outcomes serving as another confounder^55^. Furthermore, sperm DNA-fragmentation, an examination not included in the standard semen analysis, has been associated with poor reproductive outcomes in normozoospermic men^56^. External validation of this model as well as modification and further development to include the aforementioned parameters and COS cycles with dual stimulation should be the future research direction in order for this model to be clinically applicable in different settings. It should be highlighted that this single center retrospective study mainly presents qualitative data regarding the prediction values of certain parameters rather than quantitative. Further multi-center and multi-country studies, employing a wide variety of ethnicities should be performed prior to cementing the required threshold values that may be universally applicable.

Future studies should aim to robustly report on the reproductive outcomes following the double retrieval approach, and further shape LuPOR application. When comparing combination of FoPOR and LuPOR to FoPOR alone and reporting on reproductive outcomes, limited albeit encouraging, evidence originate from a single retrospective study showing a statistically significant increase in the number of oocytes retrieved and successfully fertilized following FoPOR and LuPOR, compared to FoPOR alone^18^. Large-scale prospective studies should be conducted to deeply evaluate the reproductive outcomes and conclude whether this approach should be further pursued, while defining applicability criteria. Additionally, a non-inferiority prospective trial along with a SWOT analysis should be provided in future studies, to compare the reproductive outcomes of the double retrieval within natural cycles to the conventional COS cycles. It is anticipated that the approach of double retrieval in the context of a natural cycle will lead to a lower number of oocytes retrieved compared to COS. However, it is the comparison of the reproductive outcomes that commonly guides optimal practice. In the case that future data indicates similar reproductive outcomes between COS and double oocyte retrieval following natural cycles, then the value of the double oocyte retrieval approach may be highlighted, as a means to avoid stimulation along with all that it entails. On the other hand, the double retrieval presents with a level of inconvenience for both patients and practitioners, as it requires significantly more appointments, which may be time-consuming for the patients, while considerably increasing clinic’s workload. Cost-effectiveness studies accounting for these aspects of treatment may provide more insight. Hitherto, data are lacking regarding the effect of the double retrieval on cumulative live-birth rates. Future studies should report on this outcome, as the strategy of the double retrieval requires a protocol of embryo “banking” employing collection of cryopreserved embryos prior to proceeding with an ET^31^.Thus, it may be of interest to thoroughly investigate whether the employment of multiple cycles of embryo collection and subsequent cryopreservation will lead to increased cumulative live-birth rates.

Employing double oocyte retrieval may prove beneficial for POR patients that opt to exhaust possible options prior to investigating donation. Despite the fact that a number of studies have been published thus far, double oocyte retrieval remains a matter of controversy, as high-quality evidence is lacking^22^. The present study aims to assist the practitioners in distinguishing the optimal POR population that will benefit from this approach. The parameters examined herein regarding their predictive value towards successful LUPOR application are part of the routine female infertility evaluation in the context of ART treatment. The proposed prediction model may primarily be employed for indicating poor-responders that will benefit from LuPOR. This model may indicate the specific subpopulation of POR women that will benefit from a yield of a higher number of oocytes in a single menstrual cycle by employing LuPOR. Thus, this study may assist practitioners when contemplating the optimal approach for poor responders without requiring further evaluations, that are not included in the standard operating procedures. This renders application of this prediction model cost-effective and user-friendly for both Assisted Reproduction Units and patients.

## Materials and methods

**Figure 3:**
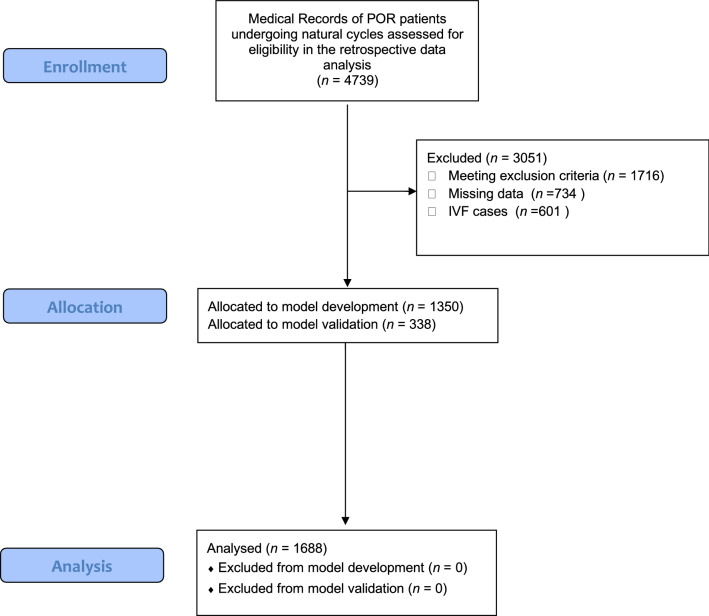
Modified version of CONSORT Flow Diagram providing a detailed outline on participants’ screening, allocation and final analysis.

A total of 4739 medical records of women undergoing natural cycle IVF, between 2012 and 2020, were screened, and 1688 medical records fulfilled the inclusion criteria for this retrospective observational study. A patients’ flowchart is presented in Fig. 3. The inclusion criteria for this study were normal ovulation, and classification of poor ovarian response according to Bologna criteria^11^. According to Bologna criteria in order for a POR diagnosis to be established, two out of the following three requirements should be fulfilled: (i) maternal age of 40 years old or above, (ii) AMH < 1.1 ng/ml or AFC < 5–7 and (iii) retrieval of 3 or less oocytes in a previous cycle with Controlled Ovarian Stimulation (COS). Only the first natural cycle performed for each couple was considered eligible for inclusion. For this retrospective analysis study, a further inclusion criterion was performance of ICSI for all couples, enabling identification of the oocytes’ maturation status following denudation of the oocytes. ICSI was performed on the grounds of male factor infertility and abnormal semen analysis parameters. Thus, only couples with POR and male factor infertility were included in the present study. This study was approved by the Genesis Athens Clinic Ethics Review Board and is in accordance to the declaration of Helsinki (Ref. No: 129/26-03-2018). Due to the retrospective observational nature of the study along with the fact that the data were obtained in an anonymized, encrypted format, the requirement for an informed consent was waived by the Genesis Athens Clinic Ethics Review Board. All patients underwent both a follicular phase oocyte retrieval (FoPOR) followed by a luteal phase oocyte retrieval (LuPOR). Women presenting with amenorrhea or cycle disorders, or other menopausal or perimenopausal symptoms, as previously described were excluded from the study^57^. Exclusion criteria for this study further included diagnosis of endometriosis, sexually transmitted diseases, current or previous cancer diagnosis, and genetic or endocrinologic disorders. Body Mass Index (BMI) above 30 or less than 18.5, Polycystic Ovarian Syndrome and Premature Ovarian Insufficiency were further considered as exclusion factors. All couples underwent basic infertility investigation.

### Basic infertility investigation

Basic infertility investigation included semen analysis, hysterosalpingography to evaluate patency of the fallopian tubes, and assessment of ovulatory function via evaluating AFC, FSH levels, LH levels, estradiol (E_2_) levels, AMH levels and progesterone’s levels during the menstrual cycle combined with ultrasonographic evaluation. The study included participants presenting with normal ovulation and reporting regular length of menstrual cycles ranging from 24 to 35 days, with less than 7 days change in cycle duration between cycles. Any abnormality observed in the anatomy of the uterine cavity or functionality of the fallopian tubes as assessed by hysterosalpingography, lead to exclusion from the present study.

### Natural IVF cycle protocol

Patients’ FSH, LH, AMH, and prolactin levels, as well as AFC were evaluated on day 2 of the menstrual cycle, along with progesterone levels evaluated on day 21 of the menstrual cycle. E_2_ was evaluated on the day of hCG administration both in FoPOR and in LuPOR.

Once the dominant follicle was identified with a diameter of ≥ 18 mm and coupled by a serum E_2_ level > 100 pg/mL, an intramuscular injection of 6500 IU of human Chorionic Gonadotropin (hCG) was administered to trigger ovulation. Following 36 h, a transvaginal ultrasonographically monitored follicular aspiration, under mild anesthesia was performed. Follicles of a diameter ≥ 13 mm were aspirated, whereas follicles of a diameter ranging between 8 and 12 mm were recorded as small follicles and were not aspirated.

The oocyte maturation status was recorded as follows: mature, referring to metaphase II (MII) stage oocyte, immature, referring to the germinal vehicle (GV) or metaphase I (MI) stage oocyte, or abnormal, based on identification of several irregular morphological characteristics with regard to oocyte shape, size, ooplasm, structure of perivitelline space, zona pellucida or polar body morphology. All oocytes were subjected to ICSI. Fertilization evaluation was performed 16–18 h post insemination, classifying oocytes as 1PN (pronucleus), 2PN, 3PN, and lysed. Normally fertilized zygotes were identified by two pronuclei and the extrusion of the second polar body. All other oocytes were classified as unfertilized, abnormally fertilized oocytes or lysed. On day 3 embryos’ morphology was evaluated according to Veeck^58^. The criteria of embryo grading according to Veeck are presented in Supplementary Table 1. Top, good, and average quality cleavage stage embryos graded as 1, 2, and 3 respectively according to Veeck et al., 1999 were vitrified on day 3. Our practice opts for either cleavage or blastocyst stage embryo vitrification as evidence suggests lack of statistical difference regarding reproductive outcomes between cleavage and blastocyst stage cryopreservation^59,60^. On the grounds of the low number of embryos resulting in the context of natural cycles, cleavage stage vitrification was preferred.

Seven days post performing FoPOR, patients were monitored for follicular growth via transvaginal ultrasonography. Women presenting with a follicle of > 13 mm diameter were monitored every two days via transvaginal ultrasonography. Criteria for practitioners to decide proceeding with LuPOR were detection of a follicle of ≥ 18 mm diameter, coupled by a serum E_2_ level > 100 pg/mL. An intramuscular injection of 6500 IU of hCG was administered in order to trigger ovulation at the luteal phase. It has been suggested that the physiological hypothalamus suppression by progesterone does not allow for an LH surge that would lead to ovulation^31^. Thus, it may be imperative to administer either hCG or Gonadotropin Releasing Hormone (GnRH) agonist triggering to enable luteal phase ovulation. Identical laboratory protocols were performed as described above, resulting to another round of cryopreserved embryo(s) originating from the same menstrual cycle. Women that underwent FoPOR but did not present with a dominant follicle of ≥ 18 mm diameter, coupled by a serum E_2_ level > 100 pg/mL did not proceed with LuPOR. Nonetheless, they were included in the analysis. The process of the double oocyte retrieval is graphically described in Supplementary Fig. 1.

### Outcome measures

The main outcome measure of the present study is the retrieval of at least one MII oocyte during LuPOR, which is herein defined as successful LuPOR application. This study aims to identify patient characteristics that may guide clinicians in decision-making when considering the practice of LuPOR. To achieve this, the authors included strictly natural cycles, to avoid possible confounders which may be associated with controlled ovarian stimulation protocols. Additionally, the rationale behind the main outcome measure being retrieval of at least one MII oocyte during the LuPOR, and not data on fertilization potential of the retrieved oocyte, is to eliminate the confounder posed by ICSI application adding another level of complexity in assessing the oocyte’s performance, as male factor infertility has been associated with poorer fertilization outcomes^55^. Oocyte retrieval, fertilization as well as successful cleavage were considered as secondary outcome measures. Moreover, it should be mentioned that the objective of this model was qualitative. To elaborate on this, this model was developed to predict retrieval of MII oocytes following LuPOR, not reporting on oocyte yield. Failure to retrieve any oocytes is usually provided as a secondary outcome in literature. However, its value is primary in the field of ART as it is synonymous to cycle cancellation.

### Statistical analysis

Statistical analysis was conducted employing the R statistical programming language through the RStudio interpreter (Boston, MA, USA). The package employed was the “cutpointr” version 1.0.32. The study aimed to associate patient characteristics with the probability of retrieving at least one MII oocyte from LuPOR, since only MII oocytes may be further subjected to insemination. Thus, the MII outcome was transformed into a dichotomous outcome representing the efficiency of LuPOR practice in resulting to retrieval of an MII oocyte–or LuPOR’s failure to result to retrieval of an MII oocyte. To achieve this, the receiver operating characteristics (ROCs) curve was calculated and the area under the curve (AUC) was employed for the determination of the predictive value of each characteristic. AUC may range between 0.5–1, with 0.5 presenting the lack of predictive value, while a value of 1 represents the perfect predictive value. The Youden’s index was employed in order to determine the threshold value. Thus, AUC of the ROC is calculated by plotting the true positive rate (sensitivity) against the false positive rate (1-specificity) across all possible thresholding values. Patients that failed to reach the follicular developmental stage of > 17 mm diameter were not subjected to LuPOR. Nonetheless they were recorded, and respective data was included in the prediction model representing retrieval of zero oocytes. Patient dataset was stratified according to age in quantiles. A random 20% of each quantile was employed to validate the model. The remaining 80% was employed to develop the model.

### Details of ethics approval

This study was approved by the Center for Human Reproduction Genesis Athens Clinic’s Ethics Review Board and is in accordance to the declaration of Helsinki (Ref. No: 129/26-03-2018).

## Supplementary Information

Supplementary Information.
