# Supplementary material for: Development of a predictive model for luteal phase oocyte retrieval in poor responders undergoing natural cycle IVF
Source: Sci Rep. 2022 May 11;12:7695. doi: 10.1038/s41598-022-11602-0 (PMC9095724; doi:10.1038/s41598-022-11602-0)
Supplement: Supplementary file 1 — Supplementary Information. [file 41598_2022_11602_MOESM1_ESM.docx]

**FoPOR**

**LuPOR**

hCG triggering

Dominant Follicle reaches 18mm

Luteal Phase

hCG triggering

Evaluation of follicular growth. If dominant follicle over 13mm proceed to LuPOR

Day 2

Dominant Follicle reaches 18mm

Follicular Phase

Evaluation of FSH, LH, AFC, AMH, Prolactin

Supplementary Figure 1: Process of double oocyte retrieval

Supplementary Table 1: Grading system according to Veeck:

| Grade | Description |
| --- | --- |
| Grade 1 | Blastomeres of equal size without fragmentation |
| Grade 2 | Blastomeres of equal size with minor fragmentation |
| Grade 3 | Blastomeres of unequal size without or with minor fragmentation |
| Grade 4 | Blastomeres of equal or unequal size with significant fragmentation |
| Grade 5 | Four or less blastomeres of any size and severe or complete fragmentation |
